# Exosomes derived from platelet-rich plasma present a novel potential in alleviating knee osteoarthritis by promoting proliferation and inhibiting apoptosis of chondrocyte via Wnt/β-catenin signaling pathway

**DOI:** 10.1186/s13018-019-1529-7

**Published:** 2019-12-30

**Authors:** Xuchang Liu, Lubo Wang, Chengshan Ma, Guozong Wang, Yuanji Zhang, Shui Sun

**Affiliations:** 1 Department of Emergency Surgery, Shandong Provincial Hospital Affiliated to Shandong University, 324 Jingwuweiqi Road, Jinan, 250021 Shandong China; 2 School of Medicine, Shandong University, 44 Wenhua Road, Jinan, 250012 Shandong China; 3 Shandong Provincial Hospital Affiliated to Shandong First Medical University, Taian, 271016 Shandong China; 4 Department of Joint Surgery, Shandong Provincial Hospital Affiliated to Shandong University, 324 Jingwuweiqi Road, Jinan, 250021 Shandong China; 5 Department of Trauma Surgery, Shandong Provincial Hospital Affiliated to Shandong University, 324 Jingwuweiqi Road, Jinan, 250021 Shandong China

**Keywords:** Platelet-rich plasma (PRP), Osteoarthritis (OA), PRP-Exos, Wnt/β-catenin, Chondrocytes, Exosomes, PRP-As

## Abstract

**Background:**

Platelet-rich plasma (PRP) provides a nonsurgical approach for treating osteoarthritis (OA). Exosomes that play vital roles in intercellular communication have been studied extensively. Here, we investigated the therapeutic potential and molecular mechanism of exosomes derived from PRP (PRP-Exos) in alleviating OA.

**Methods:**

Exosomes derived from PRP(PRP-Exos) were isolated and purified using the exoEasy Maxi Kit and then identified and analyzed. Primary rabbit chondrocytes were isolated and treated with interleukin 1 beta (IL-1β) to establish the OA model in vitro. Proliferation, migration, and apoptosis assays were measured and compared between PRP-Exos and activated PRP (PRP-As) to evaluate the therapeutic effects on OA. The mechanism involving the Wnt/β-catenin signaling pathway was investigated by Western blot analysis. In vivo, we established animal knee OA model by surgery to compare the therapeutic effect of PRP-Exos and PRP-As.

**Results:**

We successfully isolated and purified exosomes from PRP using the exoEasy Maxi Kit. We also isolated and identified chondrocytes from the New Zealand white rabbit and established the IL-1β-induced OA model; meanwhile, PRP-Exos and PRP-As both inhibited the release of tumor necrosis factor-α(TNF-α) and there was no statistically significant difference between the two. In proliferation, migration, scratch assay, the promoting effect of PRP-Exos was significantly more better than PRP-As. Furthermore, PRP-Exos could significantly decreased apoptotic rate of OA chondrocyte compared with PRP-As. In Western blot analysis, the expression of β-catenin, and RUNX2, Wnt5a were increased in IL-1β-treated chondrocytes, but PRP-Exos and PRP-As could both reverse these changes, and the reversal effect of the former was better than the latter. In vivo, we found that both PRP-Exos and PRP-As displayed the progression of OA, and the effect of PRP-Exos was obviously better than PRP-As by chondrocyte count and Osteoarthritis Research Society International (OARSI) scoring system.

**Conclusion:**

The therapeutic effects of PRP-Exos on OA were similar or better compared with those of PRP-As in vitro or in vivo. PRP-Exos acting as carriers containing growth factors derived from PRP present a novel therapy for OA by activating the Wnt/β-catenin signaling pathway.

## Introduction

Osteoarthritis (OA) is a significant health issue that is common among middle-aged and elderly populations worldwide and is associated with chronic pain, functional limitations, and economic burden [1, 2]. To date, physical therapy practices, non-steroidal anti-inflammatory drugs (NSAIDs), and intra-articular injections (IA) have been reported to alleviate the pain symptoms of patients with OA but are not conductive to alleviating the degeneration of articular cartilage [3, 4]. Recently, a new approach to the treatment of OA, IA injection of platelet-rich plasma (PRP), plays a vital role in promoting chondrocyte proliferation, differentiation, and matrix synthesis [5, 6]. Therefore, exploring the molecular mechanism of PRP in treating OA brings great significance for us.

Platelet-rich plasma is an autologous derivative of whole blood containing a higher platelet count than that of peripheral blood [7]. It has been reported that PRP can promote re-epithelization of chronic cutaneous wounds [8], enhance bone regeneration [9], augment tendon and ligament repair [10, 11], treat chronic femoral osteomyelitis [12], and prevent glucocorticoid (GC)-induced apoptosis in osteonecrosis of the femoral head (ONFH) [13]. Moreover, the regenerative and anti-inflammatory effects of OA have been widely reported [4, 14–16], and IA administration of PRP provides a nonsurgical approach for advanced-stage disease [17]. It is believed that activated platelets secrete high amounts of growth factor (GF) and cytokines, the delivery of which contributes to the major functions of PRP, including promoting proliferation and inhibiting apoptosis of chondrocyte s[18–20]. Although PRP has broad prospects for the treatment of OA, its specific molecular mechanism is still unclear. Some studies have reported that exosomes derived from PRP may be the main mechanism by which PRP treats O A[13, 18].

Exosomes are small vesicles 50–150 nm in diameter that contain specific proteins, lipids, and nucleic acids, such as DNA, mRNAs, miRNAs, and other non-coding small fragments [21, 22]. Exosomes can be secreted by almost all metabolically active cells and can be found in bodily fluids such as blood, urine, breast milk, and saliva [23–25]. They are thought to act as vehicles of bioactive lipids, proteins, mRNAs, and miRNAs and are regarded as playing vital roles in intercellular communication [26, 27]. In recent years, evidence has demonstrated the functions and underlying mechanisms of exosomes derived from various cells and extracellular fluids, such as stem cells [28], immune cells [29, 30], bone marrow stem cells (BMSCs) [31], breast cancer cells [32], and synovial fluid [33]. However, few studies have examined the functions and roles of platelet-derived exosomes [8]. In 2004, Janiszewski et al. isolated exosomes from platelets and illustrated the relationship between platelet-derived exosomes and the pathophysiology of sepsis [34]. With a deeper understanding of PRP and exosomes, Torreggiani et al. isolated exosomes from PRP [35] and first reported the role of exosomes derived from platelet-rich plasma (PRP-Exos) in tissue regeneration. Recently, PRP-Exos had been reported to have an underlying beneficial effect in preventing osteonecrosis induced by glucocorticoids [13] and promoting the re-epithelization of chronic cutaneous wounds [35].

According to these findings, PRP generally contains various kinds of growth factors after PRP activation, such as platelet-derived growth factor (PDGF), transforming growth factor-β (TGF-β), and vascular endothelial growth factor (VEGF) [7]. These growth factors contribute to tissue regeneration and cartilage repair [36, 37] and are encapsulated in exosomes to avoid destruction before arriving at the target cells [34, 38]. Moreover, exosomes have been confirmed to have low immunogenicity or tumourigenicity [39, 40]. Above all, PRP-Exos can be regarded as nano-delivery treatments and have extensive sources [38]. Nonetheless, the effects and underlying molecular mechanisms of PRP-Exos in osteoarthritis have not been reported and remain unclear.

Recently, activation of the canonical Wnt/β-catenin signaling pathway was observed in OA pathogenesis, with increased expression of β-catenin protein [41]. The expression of Wnt5a protein was also increased [42–44]. It has been reported that the Wnt/β-catenin signaling pathway can suppress IL-1β-induced cartilage degradation and inflammatory responses in chondrocytes [45] and downregulate expression of the RUNX2 gene to induce chondrocyte hypertrophy [46]. PRP-Exos-mediated activation of the canonical Wnt/β-catenin signaling pathway needs to be verified.

In this study, we aimed to determine whether PRP-Exos and PRP-As have a similar biological action in the treatment of OA in vivo or in vitro and to further verify that PRP-Exos ameliorate OA via the Wnt/β-catenin signaling pathway.

## Materials and methods

### Preparation of rabbit PRP

The whole blood was collected from New Zealand white rabbits by puncture of the central auricular artery and added to acid-citrate dextrose solution A (ACD-A) anticoagulant at a ratio of 9:1 (v/v). Then, the sample was gently agitated to mix. To separate platelets from erythrocytes and leukocytes in the plasma, the sample underwent two-step centrifugation. In brief, 10 mL of the mixture was centrifuged at 250×*g* for 10 min in a 15-mL centrifuge tube. Then, the blood was separated into three components: plasma, platelets, and leucocytes (the “buffer coat”), and erythrocytes from top to bottom. The top two layers containing platelets were transferred to a new centrifuge tube and centrifuged at 1000×*g* for another 10 min. Most of the supernatant plasma and approximately three-quarters of the platelet-poor plasma (PPP) layer was discarded, and the precipitated platelets were resuspended in the residual plasma to obtain 1 mL PR P[12, 18].

### PRP-Exos extraction

Exosomes were extracted carefully from PRP using the exoEasy Maxi Kit (cat. no. 76064). It was recommended to use only pre-centrifuged PRP. Briefly, PRP obtained as described above was centrifuged in conical tubes for 15 min at 3000×*g* at 4 °C to remove additional cellular fragments and cell debris. The cleared supernatant was carefully transferred to a new tube without disturbing the pellet, which formed a smear along the outer side/bottom of the centrifugation tube. Then, 1 volume of buffer XBP was added to 1 volume of PRP. The sample was mixed well by gently inverting the tube five times to allow the mixture to warm to room temperature. After that, we obtained a total of 16 mL of a mixture of PRP/XBP, which was added onto the exoEasy spin column and centrifuged at 500×*g* for 1 min. After discarding the flow-through, we placed the column back into the same collection tube. The above steps were repeated until the entire volume was no more than 8 mL, and the entire volume was centrifuged at 5000×*g* for 1 min to remove residual liquid from the membrane. Then, 10 mL of buffer XWP was added to the volume, and the volume was centrifuged at 5000×*g* for 5 min to remove residual buffer. Then, the spin column was transferred to a fresh collection tube, and the flow-through and collection tube were discarded. Next, 400 μL Buffer XE was added to the membrane and incubated for 1 min. The eluate was collected by centrifuging at 500×*g* for 5 min and once again was added to the exoEasy spin column membrane and incubated for 1 min. Finally, the eluate was gathered after centrifuging at 5000×*g* for 5 min. The eluate was used for isolation of exosomes by the exoEasy Maxi Kit [47–49]. All steps were performed at 4 °C. The exosomes were carefully resuspended in sterile PBS and stored at − 80 °C for subsequent experiments.

In this experiment, we designed activated PRP and PRP-Exos to observe the effects in treating OA, and each group contained the same total protein content, which was determined by the Pierce™ BCA Protein Assay Kit (Thermo Fisher Scientific Inc.) and standardized [50]. The activated PRP containing the main active constituents was named PRP-As. To observe the effect on cells in vitro, we used protein concentrations of 5 μg/mL and 50 μg/mL, and the in vivo concentrations were 10 μg/mL and 100 μg/mL [8].

### Identification of PRP-Exos

First, Particle Metrix ZetaView® nanoparticle tracking analysis (NTA) technology (Particle Metrix GmbH, Germany) was used to carefully estimate the size and concentration distribution of PRP-Exos. Second, after the exosomes were coated onto a 2 nm copper grid and stained using 2% uranyl acetate, a Hitachi H-7650 transmission electron microscope (TEM) was employed to observe the morphology of exosomes derived from PRP. Finally, Western blotting was used to examine the specific exosome biomarkers CD9, CD63, CD81, and HPS101 [13]. Activated PRP (PRP-As) was prepared to act as a control, according to the methods described by Torreggiani et al. [51].

### Chondrocyte isolation and culture

Under aseptic conditions, cartilage slices derived from the terminal of tibia and femur of 4-week-old New Zealand white rabbits, were cut into small pieces, washed with PBS three times, added to 0.25% trypsin (Gibco 25200-056) to digest for 1–2 h, and then digested with 0.2% collagenase II (Sigma V900892) in a 37 °C incubator with 5% CO_2_. After overnight digestion, the entire mixture was filtered through a 200 mesh strainer, and the filtrate was centrifuged at 190×*g* for 5 min. The supernatant was discarded, and the residue was resuspended in the remaining culture medium and plated onto T25 flasks (Corning 430639) with DMEM/F12 medium (Gibco 11320033) containing 10% FBS (Gibco C20012500BT) and 1% penicillin/streptomycin (Gibco 15140-122). The P1 chondrocytes were used for identification, and the P2 chondrocytes were used for the following experiments and preserved at − 80 °C [52].

### Alcian blue staining and immunocytochemistry of chondrocytes

The articular chondrocytes were identified by alcian blue staining. In brief, P1 chondrocytes were digested with 0.25% trypsin-EDTA and transferred onto glass cover slides. After fixing with 4% paraformaldehyde for 30 min, the chondrocytes were soaked in alcian acidizing fluid for 3 min, stained with alcian dyeing liquid for 30 min, rinsed with PBS, re-stained with nuclear solid red dye for 5 min, and washed with PBS for an additional 1 min. Then, the slides were dehydrated in absolute ethanol with different gradients and rinsed in xylene for 10 min. Finally, the samples were observed under a microscope.

The P2 chondrocytes were harvested and fixed in 4% (w/v) buffered paraformaldehyde solution for 15 min, followed by permeabilization with 0.2% (w/v) Triton X-100 for 10 min. Then, mouse anti-Col II (1:100; EMD Millipore, Cat. #MAB8887) and rabbit anti-ADAMTS5 (1:100; Abcam, Cat. #ab41037) primary antibodies were added to the fixed chondrocytes and incubated overnight at 4 °C. Then, the cells were completely washed with PBS. The corresponding goat anti-mouse IgG (H + L) cross-adsorbed secondary antibody conjugated to Alexa Fluor 488 (1:250; Invitrogen, Carlsbad, CA, USA, Cat. #A11001) and F(ab′)2-goat anti-rabbit IgG(H + L) cross-adsorbed secondary antibody conjugated to Alexa Fluor 555 (1:250; Invitrogen; Cat. #21430) were added to these cells for incubation. Finally, the cell nuclei were counterstained with DAPI (1:5000; Beyotime; Cat. #C1002) and observed with confocal microscopy (Olympus, Tokyo, Japan, BX61W1-FV1000) [53].

### Enzyme-linked immunosorbent assay

In vitro, chondrocytes were stimulated with IL-1β (10 ng/mL) in the presence or absence of different concentrations of PRP-As or PRP-Exos for 24 h. The supernatant of the cells was centrifuged and stored at − 80 °C until analysis. Tumor necrosis factor-α (TNF-α) was measured by enzyme-linked immunosorbent assay (ELISA) kits from eBioscience (San Diego, CA, USA) according to the manufacturer’s protocol [54]. The plates were read at 450 nm. The experiment was independently repeated three times.

### Proliferation of chondrocytes

The proliferation of chondrocytes was observed using a Cell Counting Kit-8 (CCK-8, Dojindo, ck04) [55]. Briefly, P2 chondrocytes at an initial density of 3000 cells/well were cultured with 100 μL of various media containing PBS (as the blank), interleukin 1 (IL-1, as a control), IL-1, and 5 μg/mL or 50 μg/mL PRP-Exos, or IL-1 and 5 μg/mL or 50 μg/mL PRP-As and were plated onto 96-well plates and cultured at 37 °C. At 24, 48, 72, and 120 h, 10 μL CCK-8 solution and 100 μL fresh culture medium was added to each well at each time point, avoiding air bubbles, and the plate was incubated for 1–4 h at 37 °C. Finally, the optical density (OD) values were measured by a spectrophotometric microplate reader (Bio-Rad 680, Bio-Rad, Hercules, CA, USA) at a wavelength of 450 nm. The survival/proliferation of chondrocytes is expressed as the value of the optical density (OD) of the test wells minus the absorbance of the blank wells.

### Apoptosis of chondrocytes

We used flow cytometry to detect the apoptosis of chondrocytes in vitro. After different treatments as described previously, the chondrocytes were harvested by centrifugation at 300×*g* for 5 min, washed twice with PBS at 4 °C, and then centrifuged at 300×*g* once for an additional 5 min. The chondrocytes were resuspended in 250 μL 1× Binding Buffer and adjusted to 1 × 10^6^ cells/mL. Then, 100 μL of cell suspension was added into 5 mL of fluid and mixed with 5 μL of annexin V/PE and 10 μL of 7-AAD. Afterwards, the sample was gently mixed at 4 °C under darkness for 15 min and analyzed by flow cytometr y[53] within 1 h of being added to 400 μL of 1× Binding Buffer.

### Migration of chondrocytes

Chondrocyte migration was assessed with Transwell cell culture chambers (Corning, 3422, diameter 8 μm). Briefly, 2 × 10^4^ cells were added into the upper chamber, with 700 μL of low serum culture medium (DMEM-F12, Gibco 11320033) containing 10% FBS (Gibco, 15240-112). Then, the same volume of “Drugs” containing PBS (as the blank), interleukin 1 (IL-1, as a control), IL-1, and 5 μg/mL or 50 μg/mL PRP-Exos, or IL-1 and 5 μg/mL or 50 μg/mL PRP were added into the lower chamber. After 24-h incubation at 37 °C, the chondrocytes were fixed with 1 mL 4% formaldehyde for 15 min at room temperature. After discarding the fixed liquid, the chondrocytes were washed twice with PBS, and 1 mL of 0.5% crystal violet was added to each well to stain the chondrocytes for 60 min. Then, the chondrocytes were washed with PBS three times. Finally, the upper surface of the Transwell filters was swabbed to remove cells. The chondrocytes were observed and counted at × 100 magnification in five randomly selected fields. The cells in five randomly selected fields at × 100 magnification were counted. The percentage of chondrocyte migration was calculated by normalizing the number of cells on the underside of the filter to the initial number of cells seeded and compared to that of the control, which was set at 100% [56].

### Scratch wound assay

The P2 chondrocytes were harvested to analyze the effect of PRP-Exos and PRP on the migration of chondrocytes by the scratch wound assay [57]. Briefly, 5 × 10^5^ cells were seeded onto 6-well plates and incubated at 37 °C overnight to maintain a confluent monolayer of cells. Each well was scratched using the spear along the ruler and washed twice with PBS to remove the free cells. The medium was then replaced with serum-free medium and “drugs,” containing PBS (as the blank), interleukin 1 (IL-1, as a control), IL-1 and 5 μg/mL or 50 μg/mL PRP-Exos, or IL-1 and 5 μg/mL or 50 μg/mL PRP, as described previously. We used an inverted microscope to monitor the wound closure of the chondrocytes at 0, 6, 12, and 24 h after the scratch. The images were obtained at the same position before and after incubation. Scratched areas were measured using ImageJ software.

### Western blotting analysis

Western blotting analysis was performed as described previously [58]. Protein from chondrocytes treated with PBS, IL-1β, IL-1β + PRP-As, and IL-1β + PRP-Exos was extracted with RIPA lysis buffer (Solarbio, Beijing, China), and the total protein concentration was determined with a BCA protein assay kit (Pierce, Rockford, IL, USA). Then, 30 μg extracted cellular protein was mixed with loading buffer, denatured in boiling water for 5 min, and separated by 10% (w/v) sodium dodecyl sulphate polyacrylamide gel electrophoresis (SDS-PAGE). After electrophoresis, the proteins were transferred to a polyvinylidene fluoride (PVDF) membrane and blocked in 5% (w/v) bovine serum albumin (BSA, Sangon Biotech, Shanghai, China) for 1 h at room temperature. The membrane was probed with Wnt5a (1:1000 dilution, Cell Signaling Technology), RUNX2 (1:1000 dilution, Cell Signaling Technology), β-catenin (1:1000 dilution, Cell Signaling Technology), and β-actin (1:1000 dilution, Santa Cruz Biotechnology Inc.) overnight at 4 °C. After washing in Tris-buffered saline with Tween-20 (TBST), the membranes were treated with the corresponding horseradish peroxidase (HRP)-conjugated secondary antibodies (1:3000 dilution, Cell Signaling Technology) at room temperature for 1 h. The excess secondary antibody was washed off by TBST, and the chemiluminescent signal was generated by the ECL imaging kit (Thermo Fisher Scientific, Waltham, MA, USA). β-actin was used as an internal control.

### Animal model and groupings

Twenty-four male New Zealand white rabbits (4-week-old, 2–3 kg) purchased from Jinan Xinjian biological technology company were utilized to establish the OA model and undergo experiments. All rabbits were randomly divided into four groups: (1) control group (without surgery; received normal saline by intra-articular(IA) injection once a week, *n* = 6); (2) OA model group (with surgery; received normal saline by intra-articular injection once a week, *n* = 6); (3) OA + PRP-Exos group (with surgery; received 100 μg/mL PRP-Exos by intra-articular injection once a week, *n* = 6); and (4) OA + PRP-As group (with surgery; received 100 μg/mL PRP by intra-articular injection once a week, *n* = 6). The OA model rabbits were anaesthetized by injection of 10% chloral hydrate (4 mL/kg) and underwent surgery of left knee, in which the medial collateral ligament and the anterior cruciate ligament (ACL) were cut off and the medial meniscus was excised [1, 53, 59]. After surgery, penicillin potassium (8 million U/kg, Shandong Lukang Biotechnology, China) was used to prevent infection by intramuscular injection for 7 days. After the operations, all of the rabbits were housed in individual cages at 21 ± 0.5 °C and allowed to move freely for 6 weeks. Soon afterwards, all of the rabbits were treated by intra-articular injection of drugs according to the previously described groups. Six weeks later, all the New Zealand white rabbits were sacrificed by injecting an overdose of air, and the left knee samples were harvested to assess cartilage degeneration and undergo follow-up experiments.

### Tissue staining, immunohistochemistry, and OARSI score

The rabbit joints were fixed in 4% buffered paraformaldehyde for 24 h after the samples were harvested, decalcified in 10% (w/v) EDTA (pH 7.4) for 30 days (d) at 4 °C, and embedded in paraffin. The samples were processed at 5 μm thickness by sagittal joint sections and stained with hematoxylin-eosin (HE). The expression of Collagen II and RUNX2 was analyzed by immunohistochemistry. The Osteoarthritis Research Society International (OARSI) scoring syste m[60] was used to evaluate the degree of cartilage destruction according to the percentage of the vertical clefts/erosion in the calcified cartilage and the recommended OA grading table supplied by Glasson et al. Chondrocyte counts at randomly high magnification and OARSI scores were used to evaluate cartilage conditions, and the higher the score, the more serious the destruction of articular cartilage with the total possible score was 24 [61].

### Statistical analysis

All data were presented as mean ± standard deviation (SD) unless otherwise stated. All statistical analyses were carried out with SPSS 20.0 (IBM Corp., Armonk, NY, USA). Statistical results were analyzed and all bar charts were constructed with GraphPad Prism version 7.0 (GraphPad Software, San Diego, CA, USA). Two-tailed Student’s *t* test or one-way ANOVA including the Tukey-Kramer post hoc test was used for normally distributed data comparison. Differences were considered statistically at a *P* value < 0.05.

## Results

### Characterization of PRP-Exos

**Fig. 1:**
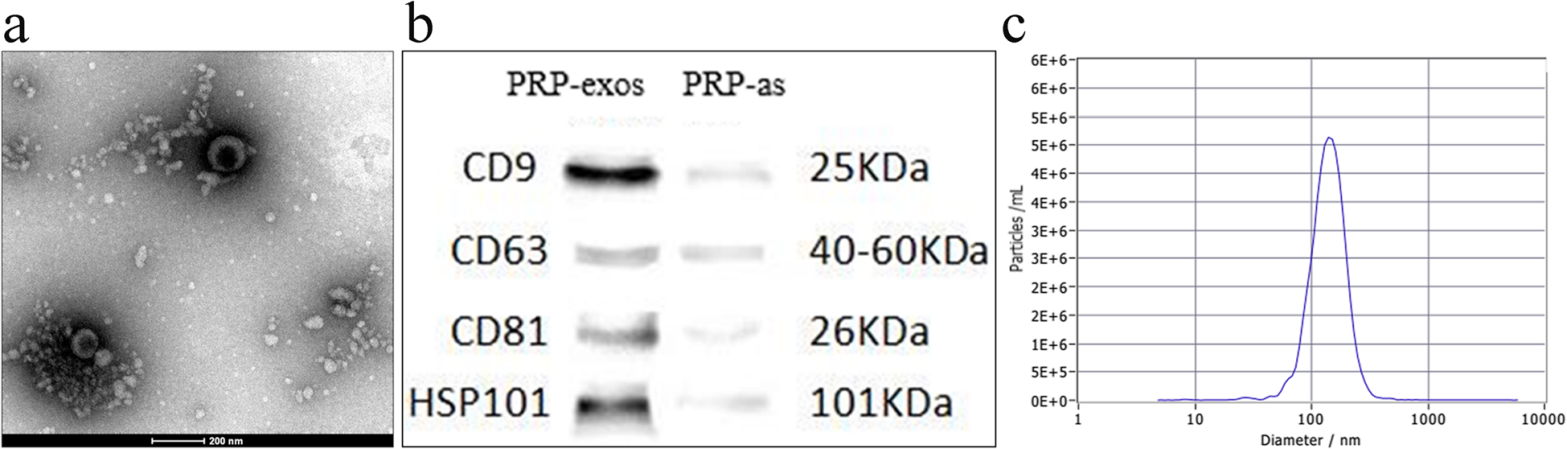
Characterization of PRP-Exos. **a** Morphology observed by transmission electron microscopy (TEM). Scale bar: 200 nm. **b** PRP-Exos surface markers (CD9, CD63, CD81, and HSP101) measured using Western Blot. Control group was the supernatant from activated PRP (PRP-As). **c** Particle size distribution of PRP-Exos measured by nanoparticle tracking analysis (NTA)

Transmission electron microscopy (TEM), nanoparticle tracking analysis (NTA) technology, and Western blotting (WB) were employed to comprehensively characterize the particles derived from PRP, called PRP-Exos. TEM clearly revealed that PRP-Exos exhibited a round-shaped morphology (Fig. 1a), and NTA showed that the majority of PRP-Exos had a similar size of 145.6 ± 50.4 nm (Fig. 1c). Additionally, Western blotting (Fig. 1b) confirmed that PRP could secrete exosomes that expressed characteristic exosomal surface markers, such as CD9, CD63, CD81, and HSP101. All data indicated that the nanoparticles derived from PRP were PRP-Exos.

### Isolation and identification of chondrocytes

**Fig. 2:**
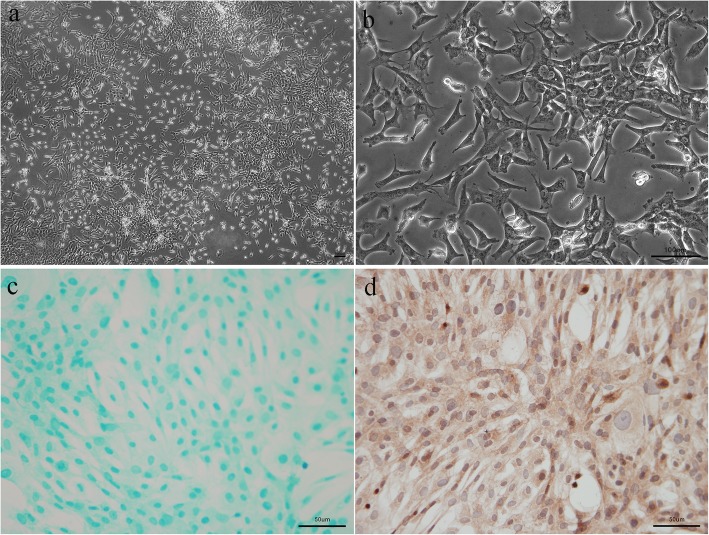
Isolation and identification of chondrocytes: The chondrocytes were obtained from New Zealand white rabbits (4 weeks). Morphology of chondrocytes observed by inverted microscope (**a**, × 40 and **b** × 200, bar 100 μm). Identification of chondrocytes performed by Alcian blue staining (**c**) and immunohistochemistry staining for Col II (**d**), bar 50 μm

The second-generation rabbit chondrocytes presented as triangles, polygons, and spindles after 24 h of culture, and the cells were gradually integrated (Fig. 2a, b). Alcian blue staining was performed to assess proteoglycan deposition in chondrocytes, which presented as blue (Fig. 2c). Immunohistochemistry staining for Col II was performed to identify that the cells we extracted were chondrocytes (Fig. 2d). The nuclei were blue, and the cytoplasm was tan. This staining showed the morphology and properties of chondrocytes.

### PRP-Exos inhibit the release of TNF-α

**Fig. 3:**
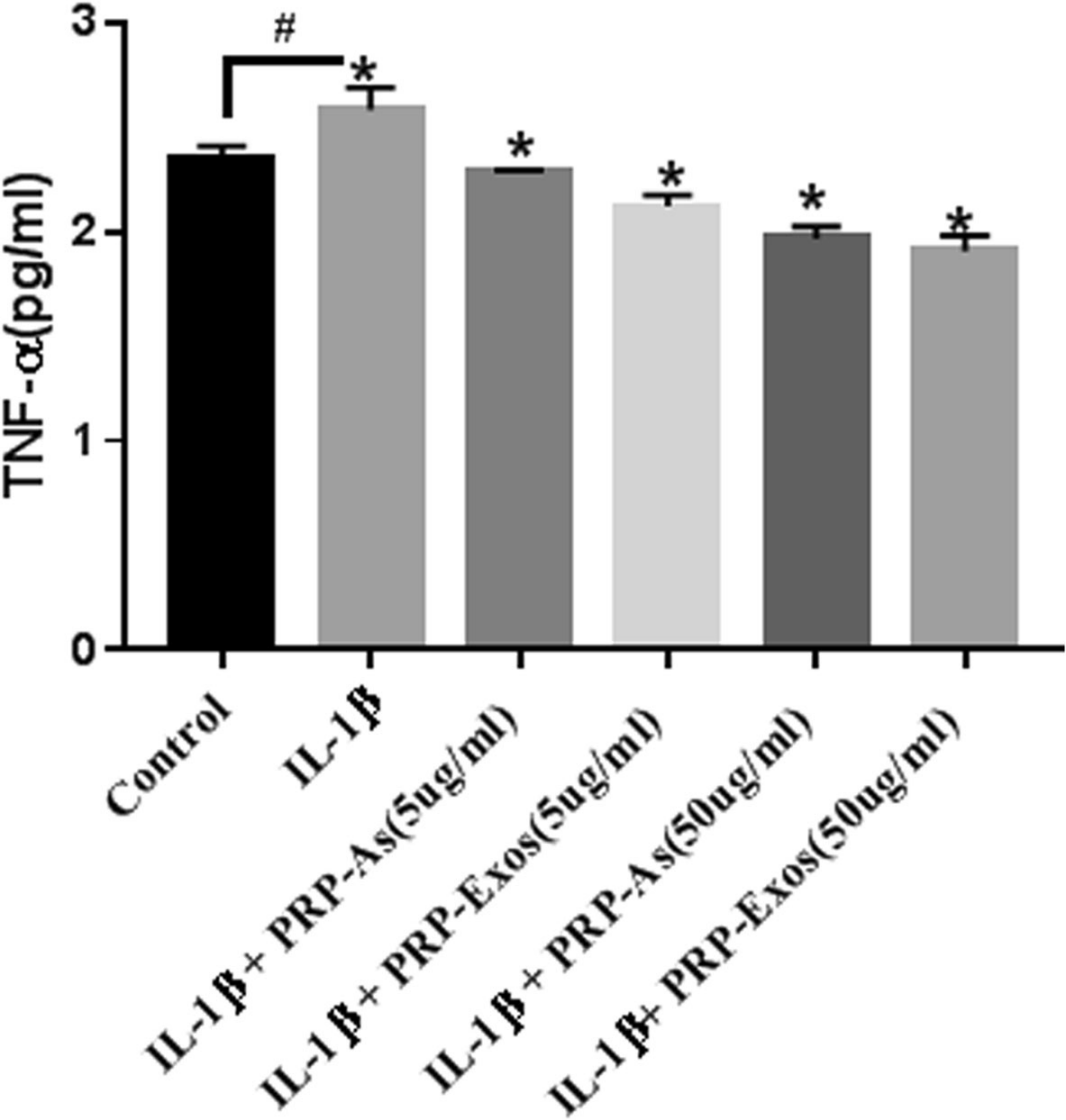
Cytokine release (TNF-α) of chondrocytes: In vitro, the chondrocytes were co-culture with PBS, IL-1β (10 ng/mL), IL-1β (10 ng/mL) added different concentration of PRP-Exos and PRP-As for 24 h. Osteoarthritis chondrocytes were established with TNF-α release increasing significantly(&<0.05). Both PRP-Exos and PRP-As with different concentration could inhibit TNF-α release significantly (*P < 0.05). Results were detected by ELISA with presenting as mean ± SEM from three independent experiments

To establish the osteoarthritis chondrocyte model in vitro, chondrocytes were incubated with IL-1β (10 ng/mL) in medium without serum for 24 h. We detected the release of the pro-inflammatory cytokines (TNF-α) into the culture medium compared with that of non-stimulated control cells by ELISA (Fig. 3). The release of TNF-α was obviously increased. Treatment with PRP-As (5 μg/mL), PRP-Exos (5 μg/mL), PRP-As (50 μg/mL), and PRP-Exos (50 μg/mL) significantly reduced the levels of TNF-α. The strongest effect was exhibited by PRP-Exos (50 μg/mL), followed by PRP-As (50 μg/mL), which was not significantly different. The inhibitory effect of PRP-Exos (5 μg/mL) was better compared with that of PRP-As (5 μg/mL).

### PRP-Exos promoted chondrocyte proliferation

**Fig. 4:**
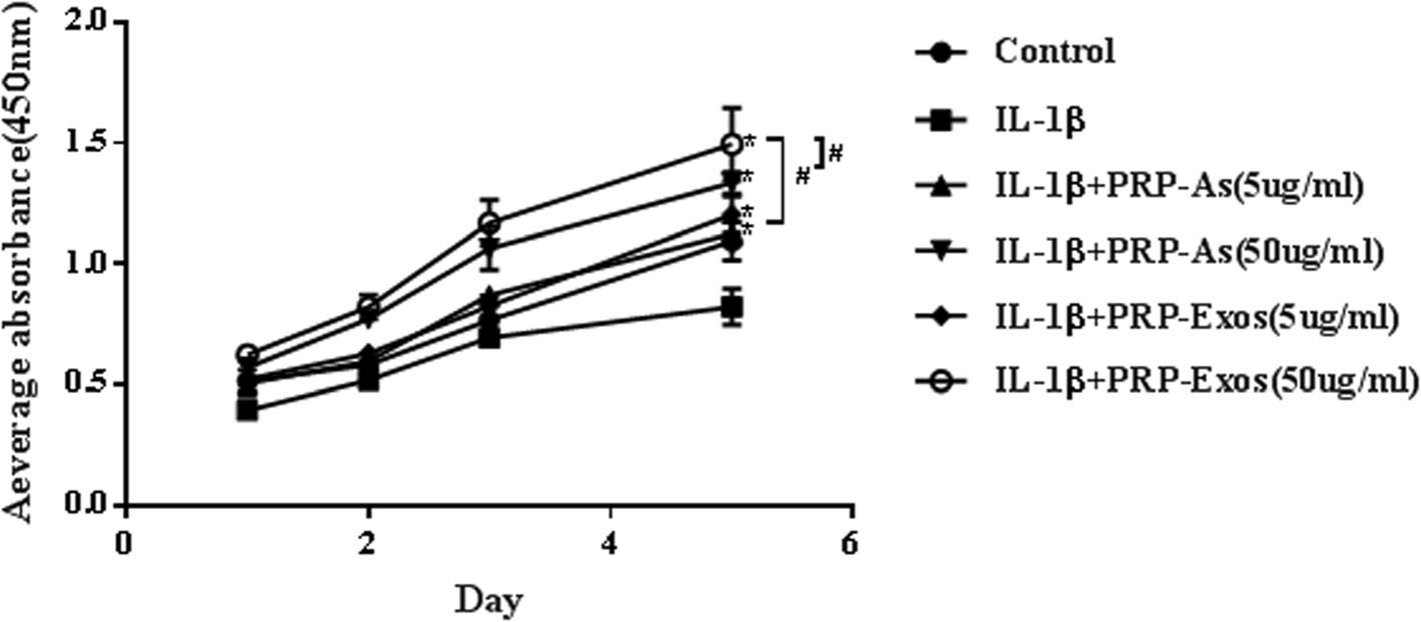
Chondrocytes proliferation. In vitro, the cell viability of chondrocytes treated with IL-1β (10 ng/mL) togehter with different concentration of PRP-Exos and PRP-As were detected at different times(24 h, 48 h, 72 h, 120 h) by CCK-8 analysis. Quantitative data were presented as means ± standard deviations of three independent experiments, *P* < 0.05. Both PRP-Exos and PRP-As significantly promoted the proliferation of osteoarthritis chondrocytes induced by IL-1β, *P < 0.05. Beyond that, the effect of proliferation stimulated by PRP-Exos compared with PRP-As in the same protein concentration was significantly better (**#***P* < 0.05)

In vitro, we explored the cell viability of chondrocytes treated with IL-1β (10 ng/mL) with different concentrations of PRP-Exos and PRP-As by CCK-8 assay. The proliferation of chondrocytes cultured for 24 h, 48 h, 72 h, and 120 h is shown in Fig. 4. Moreover, PRP-Exos and PRP-As both promoted osteoarthritis chondrocyte proliferation, and the cell viability was inhibited by IL-1β. However, chondrocytes cultured in PRP-Exos (50 μg/mL) proliferated significantly better than those cultured in PRP-As (50 μg/mL), PRP-As (5 μg/mL), and PRP-Exos (5 μg/mL) at all timepoints assessed, but the proliferation of chondrocytes cultured with PRP-Exos (5 μg/mL) showed no significant difference compared with that of the PRP-As (5 μg/mL) groups, which had similar proliferation rates.

### PRP-Exos inhibit the chondrocyte apoptosis induced by IL-1β

**Fig. 5:**
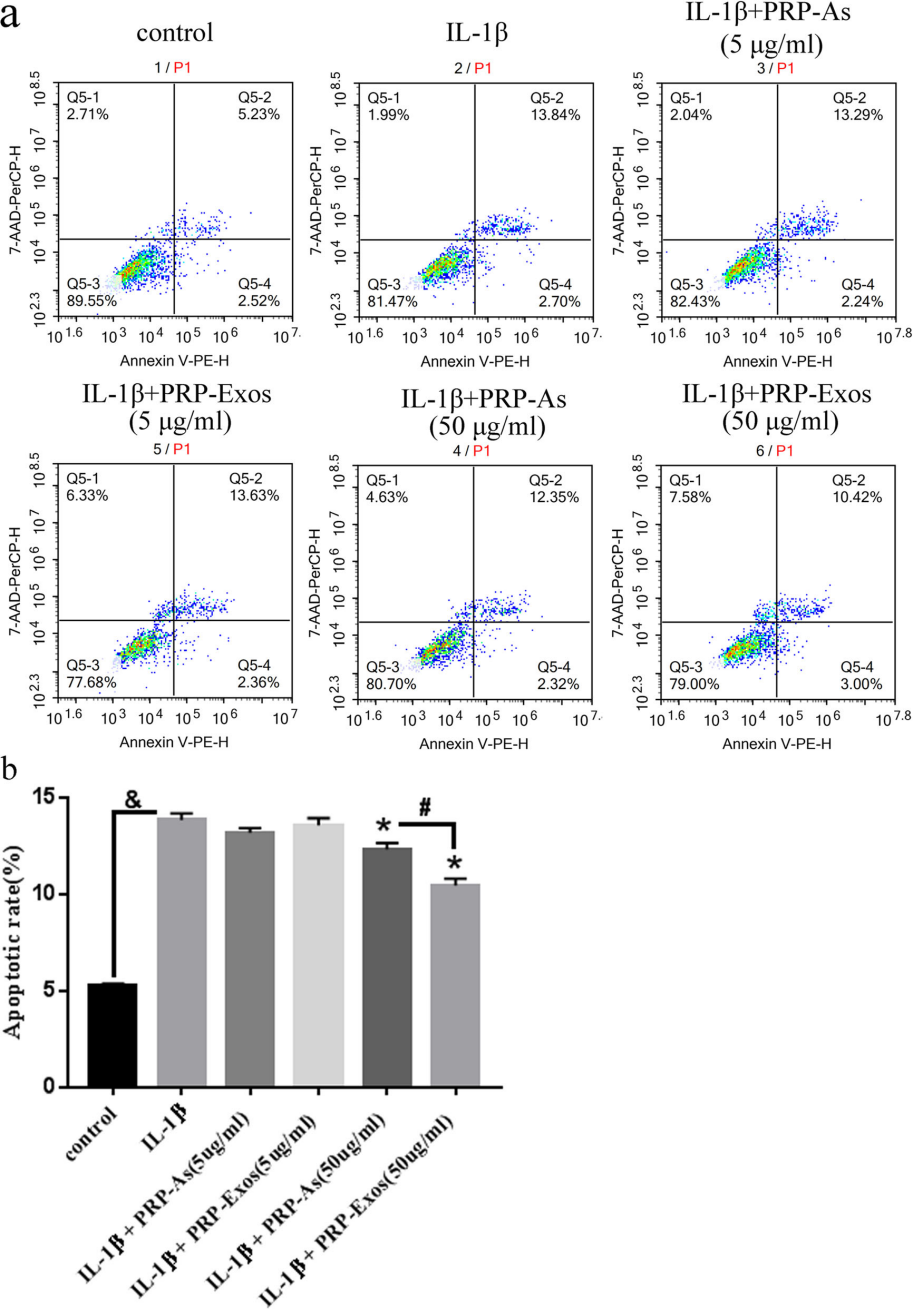
Apoptosis of chondrocytes: In vitro, the apoptosis of the chondrocytes induced by IL-1β (10 ng/mL) with or without PRP-Exos, PRP-As were assessed through Annexin V-PE-H staining with flow cytometric analysis (**a**). In vitro, IL-1β significantly increased apoptotic rate of chondrocytes (&*P* < 0.05, compared with control group). PRP-Exos and PRP-As both inhibited apoptosis of chondrocytes (*P < 0.05, compared with IL-1Β group), but the effect of PRP-Exos significantly was better than PRP-As, **#***P* < 0.05.And the low concentration of PRP-Exos and PRP-Ase either obviously inhibited apoptosis of chondrocytes (**b**)

In vitro, apoptosis of the chondrocytes induced by IL-1β (10 ng/mL) with or without PRP-Exos and PRP-As was assessed with annexin V-PE-H staining with flow cytometric analysis (Fig. 5a). The results suggest that the chondrocytes induced by IL-1β had a dramatically higher apoptotic rate than that of the control group. In contrast, the chondrocytes co-cultured with high concentrations of PRP-Exos and PRP-As inhibited the apoptotic effect of IL-1β. Furthermore, the chondrocytes treated with PRP-Exos exhibited a lower apoptotic rate compared to that of the PRP-As group (Fig. 5b).

### PRP-Exos promote the migration of chondrocytes

**Fig. 6:**
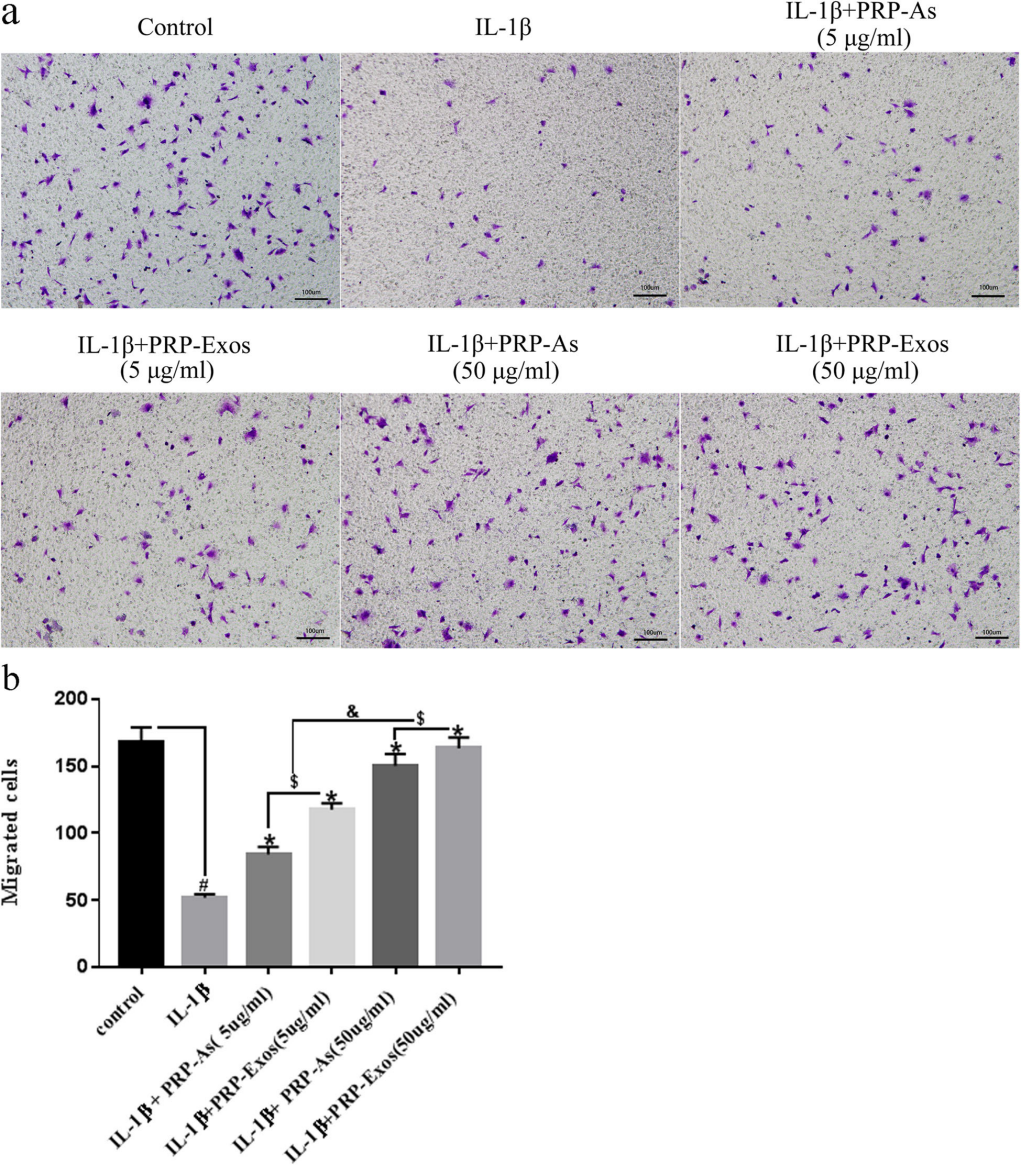
Migration of osteoarthritis chondrocytes: In vitro, the migration of osteoarthritis chondrocytes induced by IL-1β (10 ng/mL) stimulated by different concentration of PRP-Exos and PRP-As were detected by transwell assay. Scar bar: 100 μm (**a**). Both PRP-Exos and PRP-As significantly promoted the migration of osteoarthritis chondrocytes induced by IL-1β (*P < 0.05, compared with IL-1β group), the effect of migration dramatically responded to the concentration (&*P* < 0.05). Beyond that, the effect of migration stimulated by PRP-Exos compared with PRP-As in the same protein concentration was significantly better ($*P* < 0.05, **b**). Quantitative data were presented as meams ± standard deviations from three independent experiments, *P* < 0.05

As shown in Fig. 6a, IL-1β (10 ng/mL) significantly inhibited the migration of chondrocytes. However, the effect could be reversed by co-culturing the chondrocytes with PRP-Exos and PRP-As. Moreover, higher concentrations of PRP-Exos or PRP-As had a more obvious effect compared with that of the low concentration groups. Furthermore, the chondrocytes treated with PRP-Exos exhibited more migration cells compared to that of the PRP-As group (Fig. 6b).

**Fig. 7:**
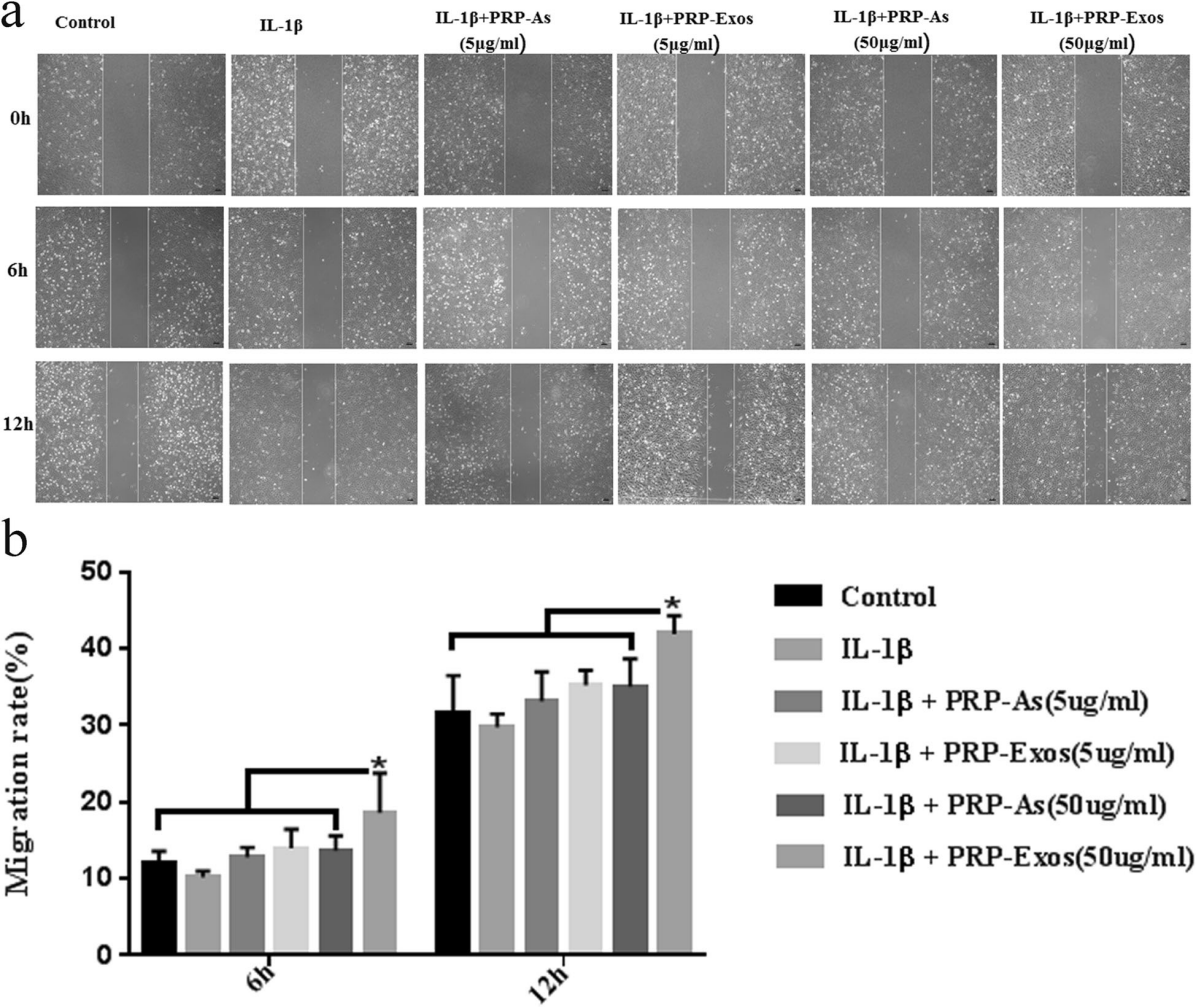
Migration rate of chondrocytes. Scratch wound assays presented the migration rate of osteoarthritis chondrocytes induced by IL-1β (10 ng/mL) stimulated by different concentration of PRP-Exos and PRP-As. Scar bar:1 00 μm (**a**). The PRP-Exos (50 μg/mL) significantly promoted the migration of osteoarthritis chondrocytes induced by IL-1β, compared with other groups (*P < 0.05), not only at 6 h but also at 12 h (**b**). Quantitative data were presented as meams ± standard deviations from three independent experiments, *P* < 0.05

Scratch wound assays (Fig. 7a) were used to detect the motility of chondrocytes and indicated that only PRP-Exos (50 μg/mL) significantly enhanced the motility of chondrocytes induced by IL-1β (10 ng/mL) at both 6 h and 12 h (Fig. 7b), and other groups did not show significant differences compared with each other.

### PRP-Exos promoted chondrocyte proliferation and migration by the Wnt/β-catenin signal pathway

**Fig. 8:**
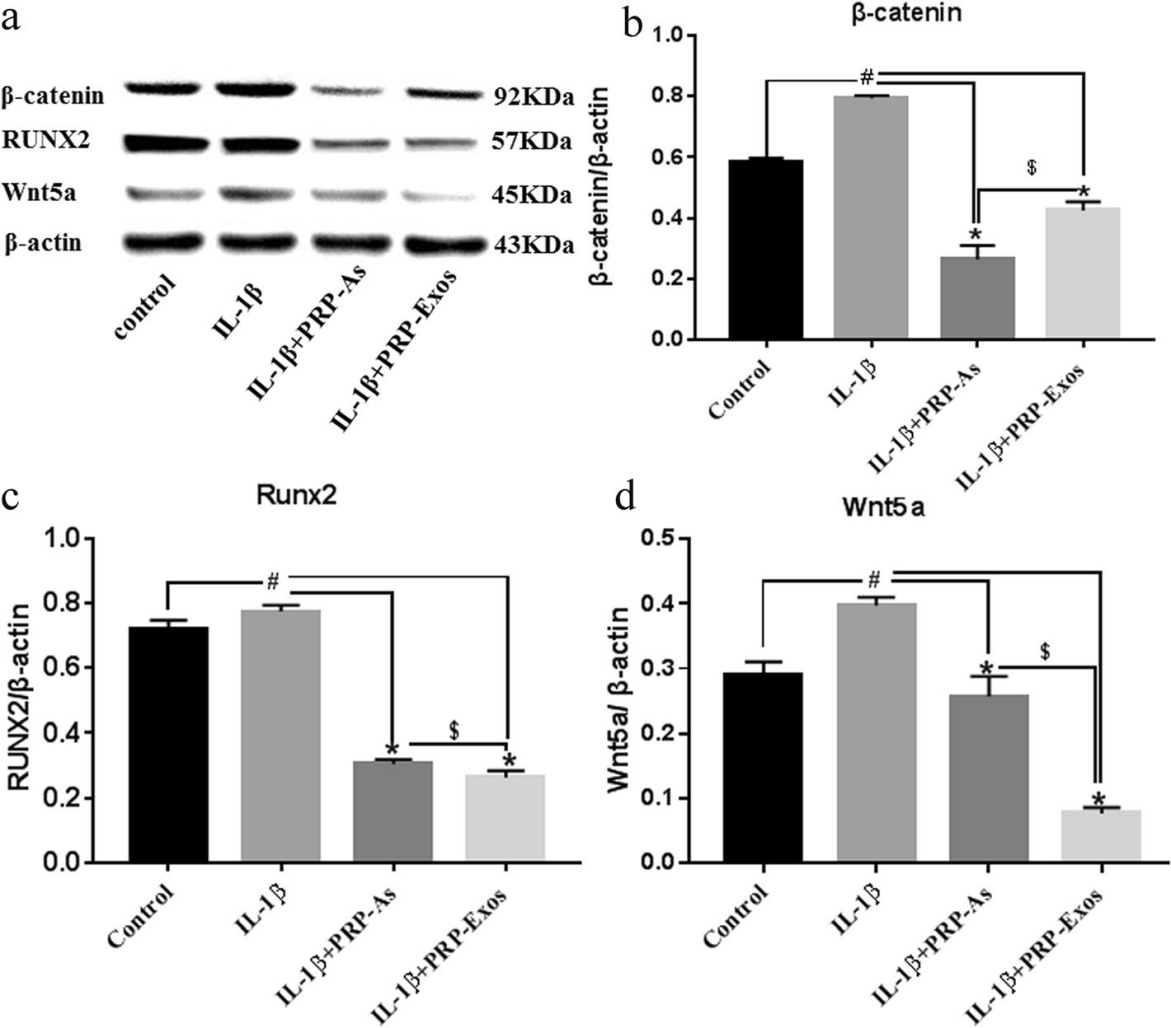
Responses of chondrocytes to stimulation by PRP-As and PRP-Exos via Wnt/β-catenin signal pathway. The chondrocytes were induced by IL-1β (10 ng/mL) stimulated and protein expression levels of β-catenin, RUNX2 and Wnt5a were estimated by Western blotting (**a**). Quantitative data are presented as means ± standard deviations of three independent experiments. β-actin was used as an internal control. Protein expression levels of β-catenin (**b**), RUNX2 (**c**), and Wnt5a (**d**) was upregulated in osteoarthritis chondrocytes, compared with control group (**#***P* < 0.05), depressed by PRP-As and PRP-Exos(*P < 0.05). The depressed effect of PRP-Exos was more obvious than PRP-As; however, protein expression levels of β-catenin was opposite ($*P* < 0.05)

To verify that the Wnt/β-catenin signaling pathway and the associated effector proteins respond to PRP-Exo stimulation, Western blotting was performed to evaluate the protein levels of β-catenin, RUNX2, and Wnt5a (Fig. 8a). In this experiment, the protein expression levels of β-catenin and Wnt5a were upregulated in chondrocytes exposed to IL-1β; furthermore, RUNX2, a downstream effector protein, was also upregulated. This result indicated that the Wnt/β-catenin signaling pathway is activated and is involved in the development of osteoarthritis. Furthermore, PRP-As and PRP-Exos reduced the protein levels to alleviate osteoarthritis. The PRP-Exo-induced inhibition was more noticeable compared with that of PRP-As for RUNX2 and Wnt5a. In contrast, PRP-As had a stronger effect on β-catenin than that of PRP-Exos (Fig. 8b–d).

### PRP-Exos prevented OA in vivo

**Fig. 9:**
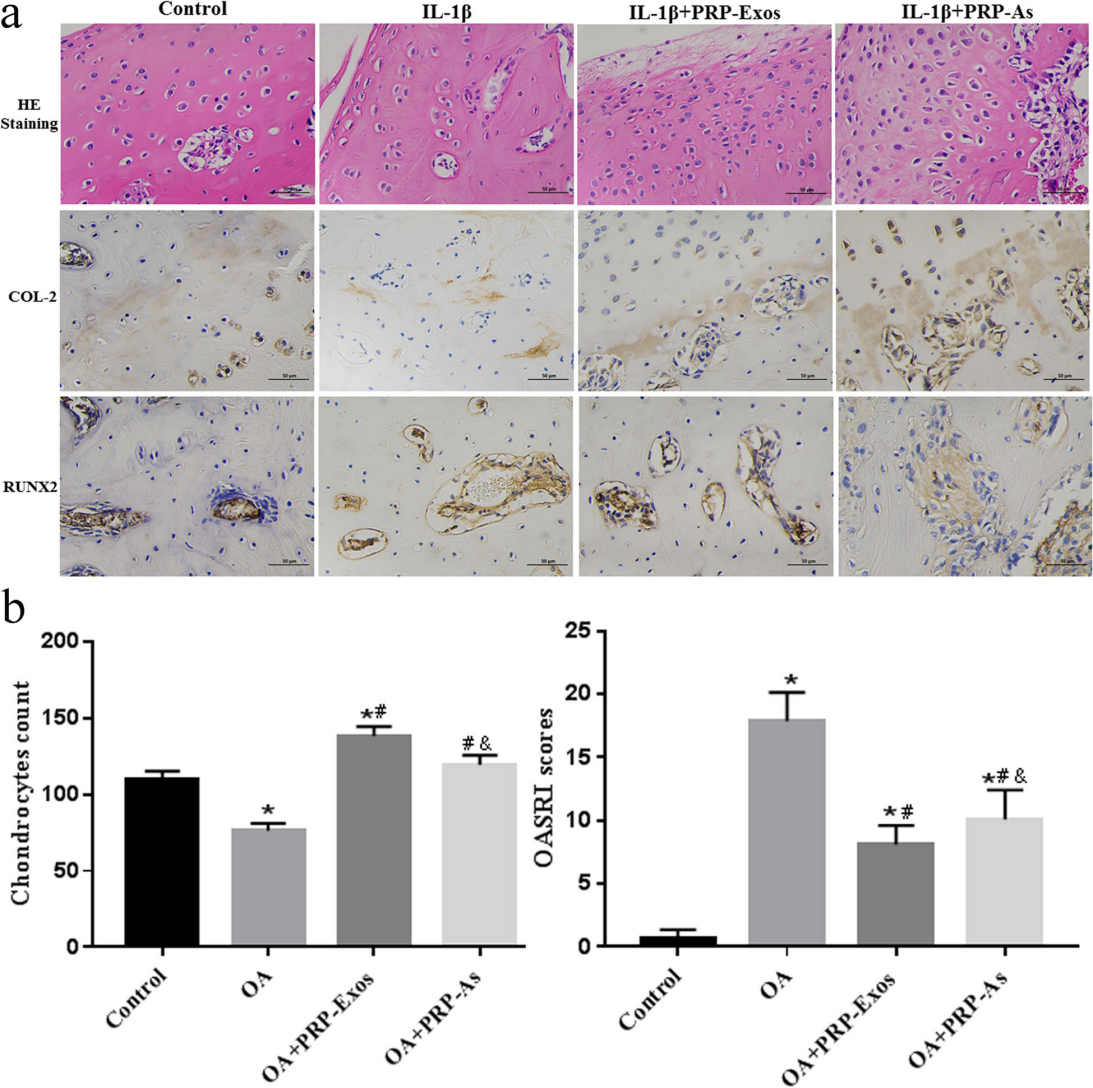
PRP-Exos prevent OA in vivo. The slides of femoral condyle (*n* = 6 for each group) were stained with Hematoxylin-eosin (HE), and the expression of *collagen* 2, RUNX2 was stained by immunohistochemistry. (Scale bar: 50 μm, **a**). The results of chondrocytes counting in randomly-selected high magnification fields (**a**) and the result of statistical analysis of OARSI score (**b**) in each group. *P < 0.05 compared to Control group. #*P* < 0.05 compared with OA group. & *P* < 0.05 compared with PRP-Exos group

In this experiment, we found that no obvious adverse events occurred, and we also confirmed the potential of PRP-Exos for OA therapy in an OA rabbit model. In the control group, a smooth surface of articular cartilage and complete structure without defects were observed, and the chondrocytes were arranged in an orderly manner with a definite boundary, and a clear tidal line was also observed. Focal hyperplasia on the surface of articular cartilage, irregularly arranged chondrocytes, and a blurred boundary were observed in the OA group. However, the abnormal phenomena in the OA group was reversed by PRP-Exos and PRP-As. The advantage of the former was obviously greater than that of the latter. Similarly, PRP-Exos and PRP-As reversed the decrease in collagen II and RUNX2 protein expression caused by osteoarthritis, promoted cartilage repair and inhibited osteoarthritis. Moreover, the effect of PRP-Exos was stronger than that of PRP-As. (Fig. 9a, b)

## Discussion

Multiple factors can result in OA, which seriously affects the quality of life of patients. The main clinicopathology of OA is destruction and degradation of articular cartilage [62], which results from the inability of adjacent chondrocytes to proliferate, migrate, and produce matrix [63]. There has been a wide range of interest in the treatment of osteoarthritis but no definite therapeutic strategy. Recently, platelet-rich plasma (PRP) and intra-articular injection have provided a potential treatment for OA. Cole BJ et al. [64] confirmed that intra-articular injection of HA (hyaluronic acid) and PRP both reduced the symptoms of OA at any time point, and the anti-inflammatory properties of PRP were obviously higher than those of HA.

In this study, we simulated the OA model of chondrocytes in vitro by exposure to IL-1β, which is one of the most prominent mediators of cartilage degradation and joint inflammation [65, 66]. It has been confirmed that the proliferation and migration of chondrocytes decreased significantly and that the apoptosis of chondrocytes obviously increased when incubated with IL-1β [52, 67]. Tofiño-Vian M et al. reported that TNF-α and IL-6 may have implications for the control of altered chondrocyte metabolism [68], and so TNF-α may be a pro-inflammatory mediator of OA. We found that TNF-α increased significantly in chondrocytes induced by IL-1β; however, both PRP-As and PRP-Exos reduced the level of TNF-α [68]. Inflammation is associated with the progression of cartilage damage in OA, and different mediators synergize to amplify and perpetuate the process. Our results showed that exosomes from platelet-rich plasma produced anti-inflammatory effects similar to those of activated PRP, which could downregulate the level of the pro-inflammatory cytokine TNF-α. Accordingly, IL-1β successfully induced OA in vitro for the following experiments.

Platelet-rich plasma (PRP) is referred to as autologous platelets, has four to five times more platelets than unprocessed blood plasma, and has been widely used in the treatment of soft tissue defects and osteoarthritis in recent years. Many experts think that growth factors released from the activation of PRP make PRP potentially effective in OA. Moreover, many studies in vivo suggest that direct injection of PRP by intra-articular injection can improve the inflammatory environment of OA patients [69]. Intra-articular injection, which is considered acceptable, non-invasive, and safe, is widely used in the clinical treatment of OA [16, 70]. Recent studies have shown that PRP might not have any direct effect on cartilage repair but contains a natural source of growth factors, which have a healing effect on cartilage damage in degenerative knee diseases [71]. Various growth factors have been detected in PRP, such as platelet-derived growth factor-AB (PDGF-AB), TGF-β1, and EGF. Among them, PDGF-AB and TGF-β1 had been confirmed previously to promote chondrocyte proliferation and cartilaginous matrix secretion in vitro [72] and accelerate cartilage regeneration in vivo [73]. Moreover, TGF-β1 could modulate the deleterious effects of IL-1β on cartilage by decreasing IL-1β receptor transcription and binding ability. Although other growth factors also have beneficial effects on cartilage regeneration, they were discovered to have concentration dependence in PRP [18]. In addition, Guo Shang-Chun et al. [33] reported that growth factors play an important role by encapsulating exosomes. In our study, we successfully separated exosomes from platelet-rich plasma (PRP), which plays an important role in OA of the knee.

Recently, a series of studies have shown that exosomes have attracted attention as new players in preventing the progression of cartilage destruction in OA. Yafei Wang et al. provided evidence that exosomes from embryonic mesenchymal stem cells (EMSCs) could modulate chondrocytes to maintain Col II expression and decrease ADAMTS5 expression under IL-1β treatment [54]. Hui Qi et al. revealed that exosomes from bone mesenchymal stem cells (BMSCs) could inhibit the apoptosis of chondrocytes and maintain chondrocyte viability under inflammatory conditions [52]. Shipin Zhang et al. showed that MSC exosomes could repair and regenerate critical osteochondral size defects by mounting a coordinated, multi-faceted response to enhance proliferation, migration, and matrix synthesis, attenuating apoptosis [74]. Exosomes are believed to provide anti-inflammatory effects by cell-to-cell communication [75]. Exosomes may directly stimulate target cells through receptor-mediated interactions or may transfer from the host cell to the recipient cell various bioactive molecules such as proteins, mRNA and miRNA [76, 77]. In the present study, although exosomes from platelet-rich plasma (PRP-Exos) were distinguished from other exosomes from different sources based on their size, they were formed intracellularly within bilayer lipid bodies and have common surface markers, such CD9, CD63, CD81, and HSP101 [78]. Surprisingly, we discovered that exosomes from platelet-rich plasma (PRP-Exos) played the same important role as PRP and were better than PRP in some aspects. PRP-Exos had a better effect in promoting chondrocyte proliferation and migration and attenuating apoptosis than PRP. In addition, PRP-Exos have been confirmed to have other roles. Shang-Chun Guo et al. reported that they successfully isolated and purified exosomes from human PRP, and PRP-Exos encapsulated bFGF, PDGF-BB, VEGF, and TGF-β; PRP-Exos increased the proliferation and migration of HMEC-1 cells and fibroblasts to a greater extent than PRP [8]. Shi-Cong Tao et al. discovered that PRP-Exos play an anti-apoptotic role against GC-ER stress-induced apoptosis in vitro and in vivo [13]. Thus it could be seen PRP-Exos play an important role in the progress of OA therapy, and may take the place of PRP.

Many factors can result in OA, such as subchondral bone changes, progressive articular cartilage degradation, osteophyte formation at the edges of the joint, inflammation and hyperplasia of the synovial tissue degeneration of ligaments and menisci (in the knee), and joint capsule hypertrophy [78]. PRP-As and PRP-Exos can reduce OA symptoms, including deformity, joint stiffness, severe pain, and limitation of joint movement with disease progression. The molecular mechanisms of these processes are still not well understood [79]. In many studies, the Wnt/β-catenin signaling pathway has been identified as possibly playing a crucial role in anti-inflammatory effects by controlling the proliferation and apoptosis of chondrocytes. Wnt proteins were reported to activate the canonical Wnt signaling cascade by acting in a paracrine manner [13] and affect cellular homeostasis by regulating cell proliferation, cell fate determination and differentiation [80]. Wnt5a has been demonstrated to increase MMP expression and inhibit collagen II expression in chondrocytes [44]. Shi-Cong Tao et al. reported that Wnt5a and Wnt5b promoted chondrocyte proliferation and migration by activating YAP through alternative Wnt signaling pathways [81]. However, others have demonstrated that Wnt5a and Wnt5b were able to inhibit chondrocyte hypertrophy via nuclear factor κB (NFκB) and JNK, respectively [82]. In the present study, we found that the accumulation of β-catenin and Wnt5a increased in IL-1β-induced osteoarthritis chondrocytes, and expression of the downstream transcription factor, RUNX2, was also increased. However, PRP-Exos and PRP-As reversed this phenomenon, and the reversal effect of PRP-Exos on Wnt5a and RUNX2 was better than that of PRP-AS, in contrast to the effect on β-catenin. Some studies had a similar conclusion; for instance, Guping Mao et al. [22] found that exosomal miR-92a-3p functions as a negative regulator by downregulating Wnt5a in both chondrogenesis and OA pathogenesis. Yazici Y et al. reported that the Wnt inhibitor M04690 is a potent inhibitor of the Wnt pathway and has potential as a disease-modifying OA drug (DMOAD) [83]. Activation of the Wnt/β-catenin signaling pathway could upregulate RUNX2 in osteoarthritis cartilage cells, and downregulation of RUNX2 could alleviate OA [46]. Therefore, PRP-Exos and PRP-As may improve OA by the Wnt/β-catenin signaling pathway.

Previous studies have shown that PRP by intra-articular injection is a potential clinical therapy for OA because there is no immunogenicity and PRP is autologous. We designed an animal experiment in which we measured the therapeutic effect of PRP-Exos by intra-articular cavity injection in a rabbit OA model based on knee joint instability induced by surgery. We found that both PRP-Exos and PRP-As improved OA of the knee joint; sometimes, the therapeutic effect of PRP-Exos was obviously better than that of PRP-As with the same treatment volume and concentration in vivo. However, the relevant molecular mechanism is not clear. Recently, PRP derivatives such as PRP-Exos have been considered novel therapies based on similar content. Although limited evidence suggests that PRP-Exos and PRP-As could modulate the Wnt/β-catenin signaling pathway [13, 44, 81, 82], further studies are needed to illuminate the mechanisms by which PRP-Exos are involved in treating OA.

In our experimental design, we simulated the environment of OA induced by IL-1β in vitro, established a rabbit OA model in vivo, and eliminated the interference of subjective and related factors as far as possible to strive for objectivity of results. However, there are still some limitations to our study. First, the detailed Wnt/β-catenin signaling pathway in PRP-Exos undergoing chondrogenesis has not yet been identified and clarified. We plan to conduct more detailed signaling experiments in rabbit chondrocytes to explore the molecular mechanisms of the signaling pathway in cartilage repair. Second, microRNAs and cyclic peptides [84] from exosomes have been reported in OA. However, the isolation from PRP-Exos and the relationship between them remains unknown and needs to be further researched. Third, the combined application of PRP-Exos and scaffolds needs to be explored in promoting cartilage defect repair in rabbits or rats. Furthermore, the optimum concentration, number, and interval of joint injections with PRP-Exos that are obtained as “autologous” drugs need to be explored.

## Conclusions

In the present study, we successfully isolated and purified exosomes from PRP. We discovered that PRP-Exos could increase proliferation and migration and decrease apoptosis of OA chondrocytes induced by IL-1β. Furthermore, the potential mechanism of PRP-Exos for OA therapy might be through activation of the Wnt/β-catenin signaling pathway. Because the most pronounced effect of PRP-Exos was better than that of PRP, or some effects were similar, PRP-Exos may play the dominating role in the progress of PRP alleviating OA. In conclusion, we suggest that PRP-Exos have a potential therapeutic effect on OA, and intra-articular injection of PRP-Exos can provide a novel approach for future exploration and application in clinical practice for the treatment of OA.

## Data Availability

The datasets used and analyzed during the current study are available from the corresponding author on reasonable request.

## Abbreviations

ACD-A: Acid-citrate dextrose solution A
BMSCs: Bone marrow stem cells
BMSCs: Bone mesenchymal stem cells
CCK-8: Cell counting Kit-8
DMOAD: Disease-modifying OA drug
ELISA: Enzyme-linked immunosorbent assay
GC: Glucocorticoid
GF: Growth factor
IA: Intra-articular injection
IL-1β: Interleukin 1 beta
NTA: Nanoparticle tracking analysis
OA: Osteoarthritis
OARSI: Osteoarthritis Research Society International
OD: Optical density
ONFH: Osteonecrosis of the femoral head
PDGF: Platelet-derived growth factor
PDGF-AB: Platelet-derived growth factor-AB
PRP: Platelet-rich plasma
PRP-As: Activated PRP
PRP-Exos: Exosomes derived from PRP
RUNX2: Runt-related transcription factor 2
TEM: Transmission electron microscope
TGF-β: Transforming growth factor-β
TNF-α: Tumor necrosis factor-α
VEGF: Vascular endothelial growth factor
